# Relative risk reduction is not enough: why cardiorenal trials should routinely report absolute risk reduction and number needed to treat

**DOI:** 10.1016/j.eclinm.2026.104258

**Published:** 2026-10-12

**Authors:** Hussein Zaitoon, Muhammad Abdul-Ghani, Ralph A. DeFronzo

**Affiliations:** Texas Diabetes Institute, University Health and Diabetes Division, University of Texas Health Science Center San Antonio, TX, USA

**Keywords:** Absolute risk reduction, Number needed to treat, Relative risk reduction, Hazard ratio, Cardiorenal outcomes, Clinical trials

## Abstract

Cardiorenal outcome trials have transformed the management of cardiovascular and kidney disease, particularly through glucagon-like peptide-1 receptor agonists and sodium-glucose cotransporter-2 inhibitors. Treatment effects are commonly communicated using relative measures, including hazard ratios and relative risk reductions. Although essential for estimating treatment effects, these measures do not directly communicate the absolute magnitude of benefit experienced by patients. Absolute risk reduction (ARR) and number needed to treat (NNT) provide complementary information by incorporating underlying event risk and duration of follow-up. The distinction is clinically important. Similar relative treatment effects can produce substantially different absolute benefits depending on baseline risk, outcome definitions, follow-up duration, and study population characteristics. Conversely, a NNT from one trial should not be used to rank therapies across studies because differences in populations and endpoints strongly influence absolute event rates. For time-to-event outcomes, ARR should be estimated from cumulative event probabilities at a specified time point, preferably using appropriate survival methods. Hazard ratios reflect a lower estimated hazard over follow-up and should not be interpreted as equivalent to reductions in cumulative risk, and differences in annualized incidence rates should not be directly converted into conventional NNT estimates. We argue that relative and absolute measures should be presented together. Routine reporting of relative treatment effects, baseline event risks, ARR, and NNT, with a specified time horizon and method of estimation, would improve transparency and clinically meaningful interpretation. This approach would help clinicians, patients, guideline committees, and policymakers understand not only whether a therapy works, but also the magnitude of benefit that may reasonably be expected.

## Introduction

Before examining cardiorenal trials specifically, it is important to define the key measures of treatment effect. Relative risk reduction (RRR) describes the proportional decrease in event risk between two groups and is calculated as (1 − risk ratio) × 100%. Absolute risk reduction (ARR) is the difference in cumulative event risks between the control and treatment groups at a specified time point. Number needed to treat (NNT) is the inverse of ARR (1/ARR) and represents the number of patients who must be treated over that same specified time period to prevent one additional event. For time-to-event outcomes, ARR and NNT should be estimated from cumulative event probabilities at a specified time point, preferably using appropriate survival methods, rather than by simply subtracting annualized incidence rates.^1^ A hazard ratio (HR) is a time-to-event measure derived from survival models (e.g., Cox regression) that estimates the relative hazard of an event over follow-up. Importantly, a HR of 0.79 indicates an estimated 21% lower hazard, assuming the proportional hazards interpretation applies, and is not synonymous with a 21% reduction in cumulative risk. These distinctions are clinically meaningful: RRR and HR estimate relative effects that are often more generalizable across populations with varying baseline risk, whereas ARR and NNT are absolute measures that depend on baseline event risk, follow-up duration, outcome definitions, competing risks, and characteristics of the specific trial population.

## Why absolute effects matter

A physician reviewing the PIONEER-6 trial^2^ sees a HR of 0.79 for major adverse cardiovascular events (MACE), corresponding to an estimated 21% lower hazard, and may reasonably perceive semaglutide as offering substantial cardiovascular benefit. However, the trial reported MACE in 3.8% of patients receiving semaglutide versus 4.8% receiving placebo over a median follow-up of 15.9 months. This corresponds to an absolute difference of approximately 1.0 percentage point and a NNT of approximately 100 over that period.^2^ The distinction is not merely statistical; it directly affects how clinicians, patients, guideline committees, and policymakers interpret therapeutic benefit.

This issue is not confined to cardiorenal medicine. In oncology, the CLEOPATRA trial demonstrated that the addition of pertuzumab to trastuzumab and docetaxel improved overall survival in patients with HER2-positive metastatic breast cancer, with a HR of 0.68. In the final analysis, median overall survival was 56.5 months with pertuzumab versus 40.8 months with placebo, a difference of 15.7 months.^3^ In an 8 year landmark analysis, overall survival was 37% versus 23%, corresponding to an absolute difference of approximately 14 percentage points.^3^ Similarly, in neurology, the AFFIRM trial of natalizumab in relapsing multiple sclerosis reported a 68% reduction in annualized relapse rate and a substantially higher proportion of patients remaining relapse free at 2 years: 67% with natalizumab versus 41% with placebo, an absolute difference of 26 percentage points.^4^ These examples underscore that the discrepancy between relative and absolute measures of effect is a pervasive concern across therapeutic areas and illustrate why both measures provide complementary information.

Cardiorenal outcome trials frequently emphasize relative effect sizes (e.g., hazard ratios) without providing the corresponding ARR, limiting clinical interpretability. Because the background incidence rate of the event studied, such as MACE or chronic kidney disease (CKD) progression, markedly influences the relationship between relative and absolute effect sizes, reporting ARR and the subsequent NNT, always specified with the corresponding time window, is critical for informed clinical decision making. Benefits expressed as RRR may appear substantial, while the corresponding absolute gains are often modest.

It is essential to recognize that relative measures of effect have distinct and important advantages. Where there is no strong evidence of effect modification, relative risk reductions are generally more generalizable beyond the trial population than absolute measures, because absolute event rates vary substantially according to baseline risk, secular trends, geography, and trial selection criteria. Guidelines appropriately use relative effects to estimate benefit across different risk strata. For an individual patient, the most appropriate estimate of absolute benefit is obtained by applying an appropriately applicable relative treatment effect to that patient's baseline risk over a specified time horizon, rather than simply relying on the trial specific ARR. Moderate to large relative risk reductions, such as the 20–39% reductions seen in several of the trials discussed here, represent substantial treatment effects even in lower risk populations and can have major effects on population level disease burden. Therefore, the argument presented here is not that ARR and NNT should supplant relative measures, but that they should be reported alongside them to provide complementary clinical perspective.

In CREDENCE, canagliflozin yielded a hazard ratio of 0.80 for MACE, corresponding to an estimated 20% lower hazard, with MACE occurring in 9.9% of patients receiving canagliflozin and 12.2% receiving placebo over a mean follow-up of 2.62 years. This corresponds to a crude absolute difference of 2.3 percentage points and an approximate NNT of 44 over the observed follow-up. In Table 1, however, the survival/model-based estimate reported at 2.5 years is an ARR of 2.5 percentage points, corresponding to a NNT of 40 (95% CI 23–165).^5^ In HARMONY Outcomes, albiglutide produced a hazard ratio of 0.78 for MACE, with events occurring in 7.1% of patients receiving albiglutide and 9.0% receiving placebo over a median follow-up of 1.6 years, an absolute difference of 1.9 percentage points and a NNT of approximately 53.^6^ Thus, these agents show broadly similar relative effect sizes when viewed through hazard ratios alone, whereas ARR and NNT reveal differences in absolute benefit that are strongly influenced by baseline event risk and follow-up duration (Table 1). These absolute differences, however, should be interpreted with caution: they may reflect differences in baseline event rates, follow-up duration, and trial populations rather than intrinsic differences between drugs. ARR and NNT are meaningful primarily within the context of a specific trial and should not be used for ranking therapies across different studies; such comparisons require appropriate adjustment for differences between trials, head-to-head trials, or formal network meta-analyses.

**Table 1:** Relative and absolute treatment effects in selected cardiorenal outcome trials.

Trial	Outcome	Composite definition	N	Events (Drug)	Events (Placebo)	Median follow-up	Event incidence (Drug)	Event incidence (Placebo)	HR (95% CI)	ARR (% points)	NNT over follow-up	Basis of ARR/NNT	Ref
PIONEER-6	MACE	CV death, non-fatal MI, non-fatal stroke	3183	61	76	15.9 mo	3.8%	4.8%	0.79 (0.57–1.11)	1.0 (over 15.9 mo)	∼100	Crude cumulative proportion (events/N); trial published a KM curve but no numerical landmark absolute estimate	2
CREDENCE	MACE	CV death, non-fatal MI, non-fatal stroke	4401	217	269	2.62 yr	9.9%	12.2%	0.80 (0.67–0.95)	2.5 (at 2.5 yr)	40 (23–165)	Trial-reported survival/model-based estimate	5
HARMONY Outcomes	MACE	CV death, non-fatal MI, non-fatal stroke	9463	338	428	1.6 yr	7.1%	9.0%	0.78 (0.68–0.90)	1.9 (over 1.6 yr)	∼53	Crude cumulative proportion (events/N); trial published a KM curve but no numerical landmark absolute estimate	6
CREDENCE	Renal composite	Doubling SCr, ESKD, renal/CV death	4401	245	340	2.62 yr	11.1%	15.5%	0.70 (0.59–0.82)	4.7 (at 2.5 yr)	22 (15–38)	Trial-reported survival/model-based estimate	5
DAPA-CKD	Renal composite	≥50% sustained eGFR decline, ESKD, renal/CV death	4304	197	312	2.4 yr	9.2%	14.5%	0.61 (0.51–0.72)	5.3 (over 2.4 yr)	∼19	Crude cumulative proportion (events/N); approximate estimate over observed follow-up	7
EMPA-KIDNEY	Kidney disease progression/CV death	Sustained ≥40% eGFR decline, ESKD, sustained eGFR <10 mL/min/1.73 m^2^, renal death, or CV death	6609	432	558	2.0 yr	13.1%	16.9%	0.72 (0.64–0.82)	5.7 (≈57/1000, end of active-trial period)	∼18 (14–23)	Trial-reported KM absolute difference (57 ± 14/1000), end of active-trial period	8
SOUL	MACE	CV death, non-fatal MI, non-fatal stroke	9650	579	668	49.5 mo	12.0%	13.8%	0.86 (0.77–0.96)	2.0 (at 3 yr)	50 (31–125)	Prespecified survival-based estimate	9

MACE, major adverse cardiovascular events; HR, hazard ratio; CI, confidence interval; ARR, absolute risk reduction; NNT, number needed to treat; KM, Kaplan–Meier; CV, cardiovascular; MI, myocardial infarction; SCr, serum creatinine; ESKD, end-stage kidney disease; eGFR, estimated glomerular filtration rate.

ARR is the between-group difference in cumulative event risk at a specified time point; NNT = 1/ARR. Both apply only to that trial's population, outcome, and time horizon. “Event incidence” columns are crude proportions (events/N), not survival estimates. Where a trial reported a survival-/model-based estimate, it is shown with time point and 95% CI: SOUL (3-year), CREDENCE (2.5-year), EMPA-KIDNEY (KM difference 57 ± 14 per 1000 at end of active-trial period; ARR ≈5.7 points, NNT ≈18). For PIONEER-6, HARMONY Outcomes, and DAPA-CKD, no landmark survival estimate was reported, so values are crude proportions unadjusted for censoring. Direct comparison of ARR or NNT across trials is inappropriate because of differences in study populations, endpoint definitions, baseline risk, competing risks, follow-up duration, and method of estimation, and trial-based ARR and NNT should not be used to rank therapies across studies.

## Interpreting ARR and NNT

The comparison of SGLT2 inhibitors for CKD outcomes warrants particular caution. Although canagliflozin (CREDENCE) and empagliflozin (EMPA-KIDNEY) showed broadly similar relative effects (HRs of 0.70 and 0.72, respectively), the renal composite endpoints differed importantly across trials. CREDENCE used a composite of doubling of serum creatinine, end stage kidney disease, and renal or cardiovascular death, whereas EMPA-KIDNEY included kidney disease progression, defined by a sustained ≥40% decline in eGFR, end stage kidney disease, sustained eGFR <10 mL/min/1.73 m^2^, or renal death, together with cardiovascular death. DAPA-CKD used a composite of ≥50% sustained decline in eGFR, end stage kidney disease, or renal or cardiovascular death.^5^^,^^7^^,^^8^ These differing definitions, eligibility criteria, baseline risk profiles, and follow-up durations influence absolute event rates and therefore ARR and NNT. EMPA-KIDNEY also enrolled a broader and, overall, lower-risk CKD population than CREDENCE. Accordingly, apparent differences in ARR or NNT across these trials should not be interpreted as evidence of differential drug efficacy.

Why does this matter? The number of cardiovascular and kidney outcome trials involving GLP-1 receptor agonists and SGLT2 inhibitors has expanded rapidly, and these therapies are increasingly being prescribed across cardiology, nephrology, endocrinology, and primary care. As treatment indications broaden, clinicians require measures that communicate not only whether a therapy works, but how much benefit patients can realistically expect.

Importantly, absolute measures of effect are often already partially available in journals that report event rates or cumulative event proportions alongside hazard ratios, from which absolute effects can be estimated. However, explicit reporting of ARR and NNT makes these calculations immediately accessible to clinicians and reduces the risk of misinterpretation. For time-to-event outcomes, the method used to estimate absolute risk is important: evidence indicates that NNTs derived from differences in incidence rates can be biased, whereas absolute risks estimated from cumulative event probabilities using appropriate survival methods provide more appropriate estimates.^1^ One area where absolute effects are particularly valuable is in comparing efficacy with safety. Relative risks alone are insufficient for balancing events caused by treatment against events prevented. The ASCEND trial, which tested aspirin versus placebo in patients with type 2 diabetes, is an instructive example: the absolute reduction in serious vascular events was 1.1 percentage points, while the absolute increase in major bleeding was 0.9 percentage points, making the balance between benefit and harm more readily apparent.^10^

The recently published SOUL trial^9^ further illustrates this point and the importance of distinguishing cumulative absolute risk from annualized incidence rates. Unlike PIONEER-6, SOUL was a larger cardiovascular outcomes trial of oral semaglutide. The hazard ratio for MACE was 0.86, corresponding to an estimated 14% lower hazard. Over the median follow-up of 49.5 months, MACE occurred in 12.0% of patients receiving semaglutide and 13.8% receiving placebo, corresponding to a crude absolute difference of 1.8 percentage points and an approximate NNT of 56 over the observed follow-up. In contrast, Table 1 presents the prespecified survival-based estimate at 3 years, with an ARR of 2.0 percentage points and an NNT of 50 (95% CI 31–125).^9^ The corresponding annualized incidence rates were 3.1 and 3.7 events per 100 person-years, respectively, an incidence rate difference of 0.6 events per 100 person-years.^9^ This latter measure is a difference in incidence rates, not a conventional ARR, and should not be converted directly into a conventional NNT by taking its reciprocal.^1^ This distinction underscores the critical importance of specifying both the time window and the method of calculation when reporting absolute treatment effects and NNT.

A fundamental limitation of NNT is that it is inherently time specific. An NNT over one year is not equivalent to an NNT over five years because treatment effects and baseline event rates may change over time. Cardiorenal outcome trials typically have follow-up durations of 2–5 years, but in clinical practice, risk modifying therapies are often used for substantially longer periods. Consequently, trial based NNT estimates should not be interpreted as estimates of lifetime benefit. Longer treatment may result in greater cumulative absolute benefit, but this cannot be assumed to increase linearly because of changing hazards, competing risks, adherence, treatment discontinuation, and other factors. For this reason, NNT should always be reported with its corresponding time window. Reporting ARR and NNT at standardized clinically meaningful time points, such as 1 year, 3 years, and 5 years when appropriate, could facilitate interpretation and reduce inappropriate comparisons across studies.

ARR and NNT themselves are not without limitations. NNT varies according to baseline risk, outcome definitions, competing risks, treatment duration, and the method used to estimate cumulative event probabilities, making comparisons across trials potentially misleading. Because trial populations are highly selected and may differ substantially from patients encountered in routine clinical practice, trial-based NNT values may not reflect the absolute benefit achievable in broader populations with different baseline risks. For time-to-event outcomes, the estimation method is also critical, and NNT should be based on absolute risks estimated at a specified time point using appropriate survival methods rather than simply on differences in incidence rates.^1^ For this reason, NNT should not replace RRR, rather, it should complement it. Reporting relative effects, ARR, NNT, baseline event rates, and the corresponding time horizon provides clinicians with a more balanced and clinically meaningful understanding of treatment effects. Recent methodological work has further explored the interpretation and potential pitfalls of NNT and related measures, reinforcing the need for caution when applying these metrics across different clinical contexts.^11^, ^12^, ^13^

## Recommendations for clinical trial reporting

The solution is straightforward. Journals should routinely require reporting of ARR, NNT, and baseline event rates alongside relative measures of effect, with the time horizon and method used to estimate absolute effects clearly stated. Clinical practice guidelines should incorporate these measures when discussing therapeutic benefits, and regulatory agencies should encourage their presentation in trial reports and prescribing information. However, it must be recognized that for population level and guideline-based recommendations, relative risk reductions remain the most generalizable measure, and the most appropriate estimate of absolute benefit for any given patient is obtained by applying an appropriately applicable relative treatment effect to that patient's individual baseline risk over a specified time horizon. Transparent communication of treatment effects is essential if clinicians and patients are to make informed decisions.

These observations illustrate that, without reporting ARR and NNT, clinicians may have difficulty appreciating the absolute magnitude of benefit when relying primarily on relative measures. Routine reporting of ARR, NNT with specified time windows, baseline event rates, and the method used to estimate absolute effects alongside relative treatment effects would enhance transparency and improve interpretation of cardiorenal outcome trials, and indeed outcome trials across all areas of medicine.

## Contributors

HZ conceived the Viewpoint and prepared the initial draft. HZ, MAG, and RAD contributed to the interpretation of the evidence, critical revision of the manuscript, and approval of the final version. All authors had full responsibility for the content of the manuscript and approved the final manuscript for submission.

## Declaration of interests

The authors declare no competing interests.
